# Multi-Level Explainable AI for ECG-Based Atrial Fibrillation Detection: Exploring LIME, SHAP, and Grad-CAM for Clinical Interpretation

**DOI:** 10.1145/3761712.3761721

**Published:** 2025-12-30

**Authors:** Jake Luo, Amirsajjad Taleban, Patrick Noffke, Rodney Sparapani

**Affiliations:** Health Informatics Program & Computer Science Department, University of Wisconsin-Milwaukee, Milwaukee, USA; Biomedical and Health Informatics, University of Wisconsin-Milwaukee, Milwaukee, USA; Engineering Department, Baxter International Inc, Milwaukee, USA; Division of Biostatistics, Medical College of Wisconsin, Milwaukee, USA

**Keywords:** Explainable Artificial Intelligence (XAI), Atrial Fibrillation Detection, ECG Analysis, Deep Learning in Cardiology, LIME and SHAP Methods, Grad-CAM Visualization, Clinical Decision Support Systems, Artificial Intelligence, Human-centered computing, Human computer interaction (HCI), Empirical studies in HCI

## Abstract

Atrial fibrillation (AFib) detection through ECG interpretation remains a critical yet complex task in clinical settings. While deep learning models have shown promise in automated ECG analysis, their ”black box” nature limits clinical adoption. This study presents a multi-level explainable AI (XAI) framework for AFib detection, combining LIME, SHAP, and Grad-CAM approaches to provide clinically meaningful interpretations. Using the PhysioNet AFib Dataset with 5,830 ECG samples, we developed a ResNet-based model that achieved 91.3% precision and 88.7% recall for AFib detection, and 98.4% precision and 98.8% recall for normal rhythm classification. While traditional LIME and SHAP analyses provided feature importance values, their point-wise granularity proved too fine-grained for clinical interpretation. We addressed this limitation by introducing a novel RR-interval aggregation method that aligns XAI outputs with clinical diagnostic patterns. Further examination using Grad-CAM successfully localized three key morphological features: P-wave absence, pronounced T-waves, and irregular RR-intervals. This comprehensive XAI framework bridges the gap between AI predictions and clinical interpretability, offering both beat-level and temporal pattern analysis that aligns with established diagnostic criteria for AFib. Our results demonstrate that integrating multiple XAI approaches can provide clinically relevant explanations while maintaining high diagnostic accuracy.

## 1 Introduction

**Figure 1:**
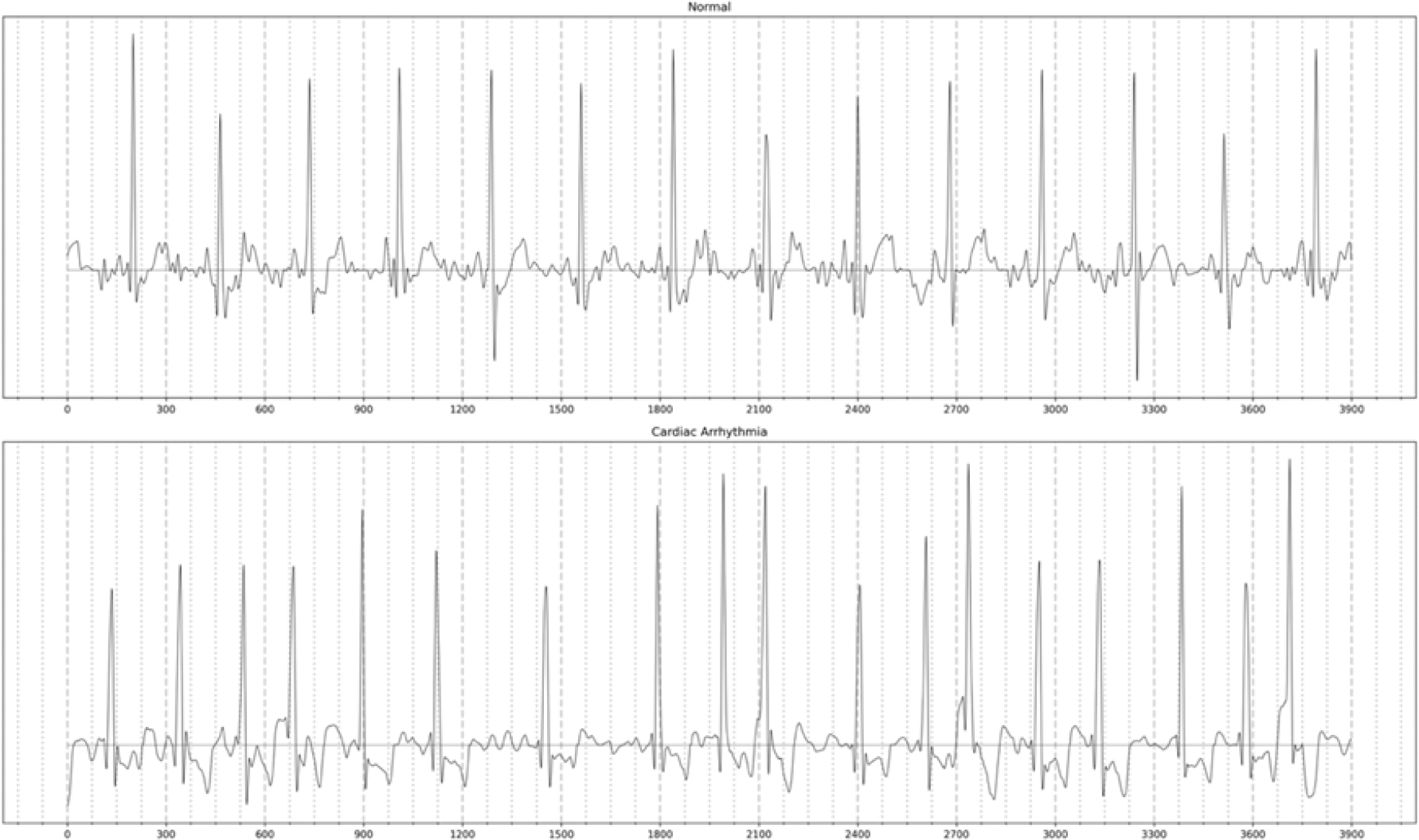
Sample Electrocardiograms (ECGs). Re top diagram shows a normal patient case, and the bottom diagram shows a patient with cardiac arrhythmia.

Cardiac arrhythmias are a significant contributor to sudden cardiac death, accounting for nearly 50% of cardiovascular-related fatalities globally [1]. The mechanisms behind arrhythmias often involve abnormal electrical impulses or conduction issues within the heart, leading to potentially life-threatening conditions such as atrial fibrillation (AFib) [2]. Electrocardiograms (ECGs) play a crucial role in detecting arrhythmias (See Figure 1), because ECGs provide a real-time visualization of the heart’s electrical activity, revealing the characteristic irregular rhythm, allowing clinicians to diagnose the condition with clinical evidence [3]. However, the complexity of ECG interpretation often complicates effective diagnosis [4, 5].

Traditional ECG analysis is a labor-intensive process that requires significant clinical expertise and time investment from healthcare providers. Cardiologists and trained medical professionals must carefully examine multiple ECG leads, identify subtle pattern variations, and interpret complex waveforms to make accurate diagnoses. This manual interpretation process can create bottlenecks in patient care, particularly in high-volume clinical settings or resource-limited environments where specialist expertise may be limited [6]. Additionally, the subjective nature of visual ECG interpretation can lead to inter-observer variability, potentially affecting diagnostic accuracy and consistency [5, 7].

Recently, the application of artificial intelligence (AI), particularly through deep learning [8], has transformed the landscape of medical diagnostics by improving accuracy. For example, a study developed a fully integrated computer-aided diagnosis system for digital X-ray mammograms, utilizing deep learning for detection, segmentation, and classification, which significantly enhanced diagnostic accuracy in breast cancer detection [9]. Deep learning algorithms also have been applied to medical image analysis, demonstrating high performance in tasks such as lung cancer detection [10]. Additionally, multi-modal medical data fusion based on deep learning effectively extracts and integrates patient characteristic from different modes, improving clinical applicability in diagnosis and medical evaluation [11]. Furthermore, deep learning techniques have been utilized in the automated analysis of medical diagnostic videos [12], such as ultrasound and endoscopy, enhancing the efficiency and accuracy of diagnosis. These advancements demonstrate the impact of deep learning on medical diagnostics, offering more accurate and efficient tools for disease detection and management.

Similar to the advancements in medical diagnostics, deep learning has significantly enhanced electrocardiogram (ECG) analysis, particularly in detecting and classifying cardiac arrhythmias. By leveraging large datasets and sophisticated algorithms, AI-driven models can identify subtle patterns in ECG signals that may be challenging for human interpretation, leading to earlier and more accurate diagnoses of conditions such as atrial fibrillation. For instance, a study developed a deep learning model that achieved cardiologist-level arrhythmia detection in ECGs, demonstrating the potential of AI in this domain [13]. In another case, AI-enabled ECG algorithms have been shown to predict stroke, cognitive decline up to 10 years before clinical diagnosis [14]. This integration of deep learning into ECG analysis not only enhances diagnostic precision but also facilitates continuous monitoring and personalized treatment strategies, thereby improving patient outcomes in cardiovascular care.

**Figure 2:**
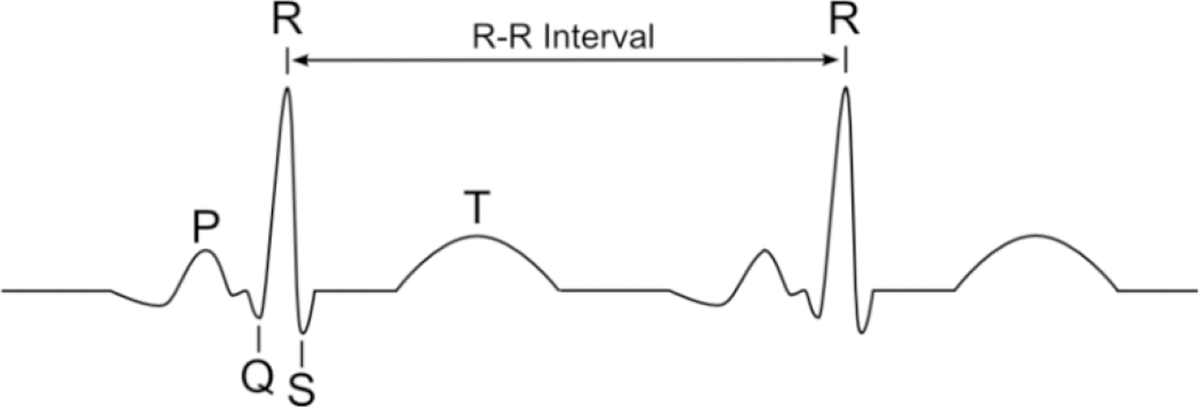
Key ECG characteristics, including R peaks, RR interval, P wave, T wave, and QRS complex. (From Wikipedia)

While deep learning has significantly advanced ECG analysis, the inherent ”black box” nature of these models presents a critical challenge in clinical settings [15]. Clinicians require more than just a diagnostic label; they need to understand the underlying reasons behind an AI’s decision to effectively plan treatments and communicate with patients [16]. This need for transparency is crucial for building trust in AI-driven diagnoses, especially when patient outcomes are at stake. Explainable Artificial Intelligence (XAI) addresses this challenge by providing insights into the decision-making processes of AI models, enhancing their interpretability and trustworthiness [17]. For instance, XAI techniques could be developed to highlight the specific ECG features (Figure 2, e.g., P-wave morphology, QRS complex duration) that contribute to an AI’s diagnosis of atrial fibrillation, allowing clinicians to validate the AI’s findings against their own medical knowledge. Moreover, XAI can help identify potential biases or errors within AI models, ensuring alignment with established medical practices and ethical standards [18].

Recent advances in XAI techniques, particularly model-agnostic approaches such as the Local Interpretable Model-agnostic Explanations (LIME) [17] and SHapley Additive exPlanations (SHAP) [19], have shown promising results in medical imaging interpretation [20]. Additionally, Gradient-weighted Class Activation Mapping (Grad-CAM) [21] has emerged as a powerful visualization technique that uses the gradients of any target concept flowing into the final convolutional layer to produce a coarse localization map highlighting important regions in the input for prediction. These methods can provide clinicians with visual explanations of which ECG segments and features most strongly influence the model’s predictions, helping to identify potential biases and align AI-assisted diagnostics with established medical knowledge [22]. XAI also could enhance the clinical adoption of AI systems by providing evidence-based justifications for model predictions, thereby supporting regulatory compliance and medical-legal requirements in healthcare settings [23]. While recent studies have explored the application of XAI techniques like LIME, SHAP, and Grad-CAM in ECG analysis, there remains a crucial gap in translating these methods into clinically meaningful interpretations [24–26].

In this study, we aim to bridge the existing gap between advanced AI diagnostics and their clinical applicability. This research is vital as it targets the transparency of automated decision-making processes in ECG analysis, which could lead to more reliable and interpretable diagnostic tools, ultimately aiming to reduce mortality associated with atrial fibrillation.

This paper makes several contributions to the field of AI-assisted cardiac diagnosis. First, we introduce a novel multi-level XAI framework that bridges the gap between AI predictions and clinical interpretability in ECG analysis. Our key innovation lies in the development of an RR-interval aggregation method that transforms granular LIME and SHAP importance values into clinically meaningful beat-level interpretations, directly addressing the limitation of traditional XAI approaches in ECG analysis. Second, we demonstrate the complementary nature of different XAI techniques by integrating LIME, SHAP, and Grad-CAM to provide comprehensive interpretations at both the temporal and morphological levels. The Grad-CAM implementation specifically reveals clinically relevant features such as P-wave absence and T-wave abnormalities, validating our model’s learning against established diagnostic criteria. Finally, our approach achieves high diagnostic accuracy (91.3% precision for AFib) while maintaining clinical interpretability, providing a practical solution for the deployment of AI in cardiac diagnosis. These contributions collectively advance the field by demonstrating how AI explanations can be aligned with clinical diagnostic workflows, potentially accelerating the adoption of AI tools in cardiac care.

## 2 Methods

### 2.1 Data Source and Extraction

We extracted data from the PhysioNet Dataset [27]. This data set is designed to enable the research community to develop machine learning models that can classify single-lead ECG recordings for AFib detection. These ECG recordings range from 30 to 60 seconds, The original data were categorized into labels: ‘normal rhythm’, ‘atrial fibrillation’, ‘alternative rhythm’, and ‘noisy’. The whole data set includes 8,528 training and 3,658 test single-lead ECG recordings, taken using AliveCor devices at a 300 Hz sampling rate and were bandpass filtered. For our analysis, we focused exclusively on normal rhythm and atrial fibrillation, excluding other classifications due to their less frequent clinical significance. This results in 5,830 ECG samples for training and testing.

### 2.2 Data Denoising and Preprocessing

Raw ECG data inherently contains a lot of noise. This could be a barrier for developing machine learning algorithms using ECGs. To address this challenge, we developed a new data preprocessing pipeline to prepare the data for machine learning tasks. The processing begins with signal denoising using a wavelet transform method [28]. This method utilizes wavelet decomposition to segment the signal into various frequency bands, effectively isolating and removing noise components. Following denoising, an adaptive Kalman filter [29] is used for further refinement of the signal. This filter applies to a series of median filters sequentially, reducing random noise while preserving the signal’s essential characteristics. The widths of these filters are dynamically calculated based on a predefined set of durations and a basic sampling rate of 300 Hz, ensuring an odd number width to maintain symmetry in the filtering process. These filters are adjusted to ensure optimal noise reduction and are based on the signal’s specific properties.

### 2.3 Deep Learning Model Development

We developed a new deep learning model to learn from the ECG data by adopting and customizing the ResNet architecture [30]. Our model features multiple convolutional layers designed to extract detailed features from input data. The convolutional layers incorporate batch normalization and activation functions to stabilize neuron activities and introduce non-linearity, respectively. ResNet blocks are used repetitively throughout the model to prevent the vanishing gradient problem and enhance training efficiency. The model’s flexible design allows it to be adapted to different types of input data and classification tasks, making it especially suitable for the precision required in medical diagnostics.

**Figure 3:**
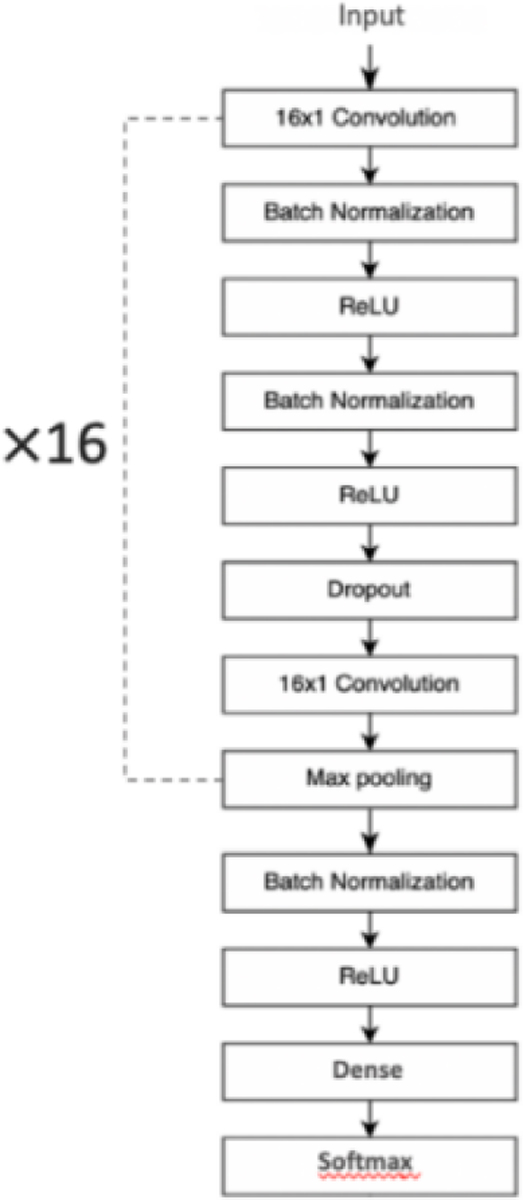
Deep learning model for AFib prediction.

In our neural network architecture (Figure 3), the ResNet block is used 16 times, serving as a fundamental unit for constructing a deep network capable of circumventing the vanishing gradient problem through its shortcut connections. These blocks enable the training of deeper networks by learning identity functions, ensuring that each additional layer contributes to performance gains. The repetition of the ResNet block is a testament to its efficacy in deep learning, allowing each successive block to build upon the refined feature maps of its predecessors. This configuration not only enhances feature extraction but also maintains training efficiency, a critical innovation that our architecture leverages to achieve state-of-the-art results.

### 2.4 Exploratory XAI Development for ECGs

To generate clinically relevant interpretations of our deep learning model for AFib prediction, we employed exploratory XAI methods, specifically LIME and SHAP. These XAI techniques were applied to the ECG data to elucidate which features were influential in classifying the ECG signals as either normal or AFib. The goal was to bridge the gap between the complex black-box nature of the deep learning model and the clinical interpretability required for medical decision-making.

We started by applying LIME and SHAP directly to the ECG time-series data produced by our trained deep learning model. LIME approximates the model’s behavior by generating surrogate models to highlight the importance of input features. In our case, LIME was used to quantify the contributions of individual ECG data points. Specifically, the LIME technique perturbed the ECG input samples by adding small amounts of noise and observed the changes in model predictions. The importance values for each input feature (ECG data point) were then derived based on the impact of the perturbations.

Similarly, SHAP values were computed to determine the contribution of each feature to the final model output. SHAP values provide a unified measure of feature importance that satisfies properties like consistency and local accuracy. For an ECG input, the model prediction can be approximated by a linear combination of Shapley values corresponding to each feature.

One of the challenges with directly interpreting LIME and SHAP results in the context of ECG analysis was that the generated importance values were too fine-grained for practical use by clinicians. To address this limitation, we proposed a novel aggregation approach that groups these importance values over clinically meaningful segments of the ECG. Specifically, we aggregated the LIME and SHAP values across RR intervals (Figure 2), which are the intervals between consecutive R-peaks in the ECG. The RR interval is clinically relevant as it reflects the timing between heartbeats, providing insights into cardiac rhythm irregularities. By accumulating LIME or SHAP values for all data points within an RR interval, we generated a composite score for each interval, effectively transforming point-wise importance into beat-level insights. This aggregation allowed us to create an intuitive visualization that highlighted segments of the ECG most indicative of AFib.

We developed visualizations that depicted the aggregated importance values across multiple RR intervals. These visualizations showed regions of the ECG in different colors, with deeper shades of red indicating stronger evidence for AFib and blue regions suggesting normal rhythm characteristics. R-peaks were marked with yellow markers to provide reference points for each heartbeat, enhancing the interpretability of the visualization. We also extended the analysis by applying Gradient-weighted Class Activation Mapping (Grad-CAM) to the model’s convolutional layers to provide spatial localization of the most influential features within the ECG beats. Grad-CAM calculates the gradients of the output class score concerning feature maps and uses them to produce a heatmap that indicates the importance of different regions in the ECG signal.

## 3 Experiment Results

### 3.1 Neural Network Prediction Performance

In our efforts to build a robust deep learning model for atrial fibrillation prediction using ECG, we utilized the PhysioNet AFib dataset comprising 5,830 ECG samples, categorized into two primary classes: normal rhythm and atrial fibrillation. To ensure both comprehensive training and robust evaluation, the dataset was randomly divided into training and testing subsets for 10-fold cross validation. Specifically, 90% of the samples (5247) were randomly selected for training, while the remaining 10% (583) were reserved for testing. This approach balanced the need for effective model training with the capacity to validate its real-world applicability. The model was trained for 80 epochs in the Medical College of Wisconsin’s Research Computer Cluster with 4 Nvidia V100 GPUs.

The trained deep learning model has 10,473,378 parameters and its performance was rigorously assessed in metrics of precision, recall, and F1-score, as summarized in Table 1. The performance of our deep learning model is evidenced by the precision metrics obtained through extensive testing. Employing a confusion matrix, we validated the model’s efficacy in distinguishing between normal heart rhythms and atrial fibrillation events, achieving a precision of 98.4% for normal rhythms and 91.3% for AFib events. The model demonstrated high reliability with a recall of 98.8% for normal and 88.7% for AFib rhythms, resulting in F1-scores of 98.6% and 90.0%, respectively.

**Figure 4:**
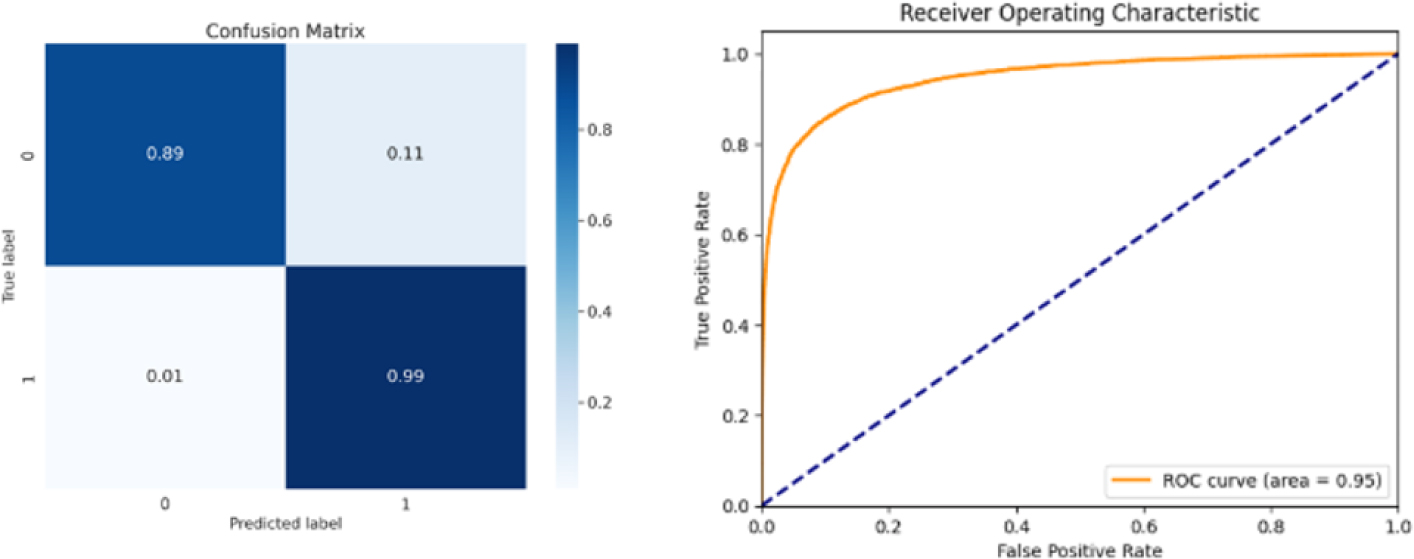
Confusion matrix and ROC curve

These metrics show that the model has a strong capability in distinguishing normal events and AFib events. The model achieves high precision and recall, particularly for normal rhythms, while maintaining commendable performance in identifying AFib cases. The built model performance is on par with other similar studies [31–33]. For a more detailed breakdown of predictions, the confusion matrix and ROC curve is presented in Figure 4.

### 3.2 Exploring XAI Analysis for AF Prediction

**Figure 5:**
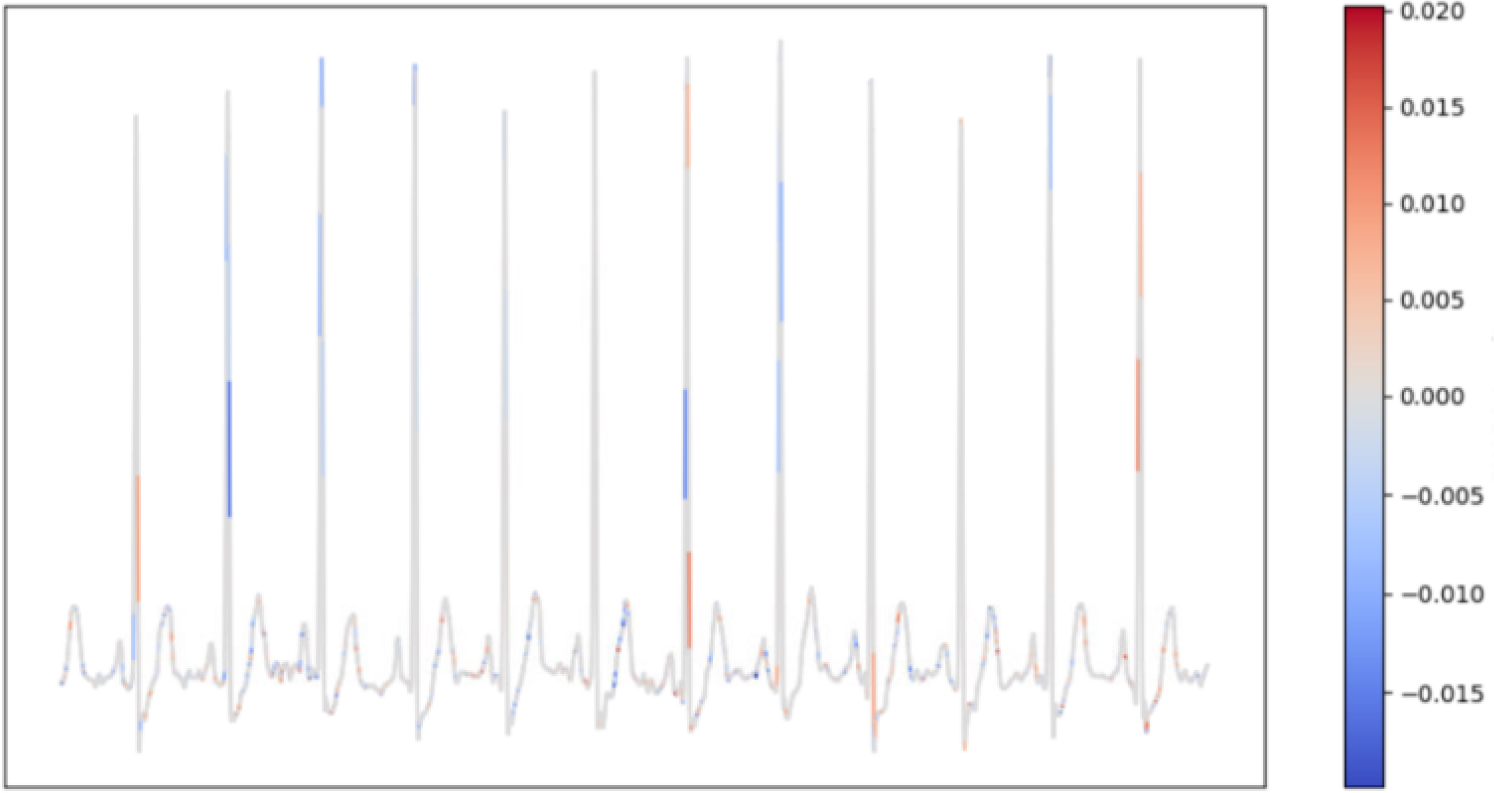
Learned LIME values visualized on ECG data.

When applying LIME and SHAP to the processed ECG data, both XAI methods successfully assigned importance values to signal components that contributed to the classification decisions. However, we identified two significant limitations in their clinical interpretability (Figure 5). First, the granularity of the importance values was too fine-grained for practical clinical interpretation. While the models assigned significance scores to individual data points along the ECG timeline, clinicians typically diagnose AFib by examining broader morphological patterns and rhythmic characteristics across multiple beats, rather than point-by-point signal variations. For instance, where LIME and SHAP highlighted hundreds of individual points of importance across a 30-second ECG strip, cardiologists instead look for patterns such as P-wave absence, irregular RR intervals, and QRS complex morphology changes across multiple beats. Second, these traditional XAI approaches did not effectively capture beat-level characteristics that are crucial for AFib diagnosis. The temporal sequence and relationship between consecutive heartbeats, which are fundamental to identifying arrhythmias, were not adequately represented in the importance values generated by LIME and SHAP.

**Figure 6:**
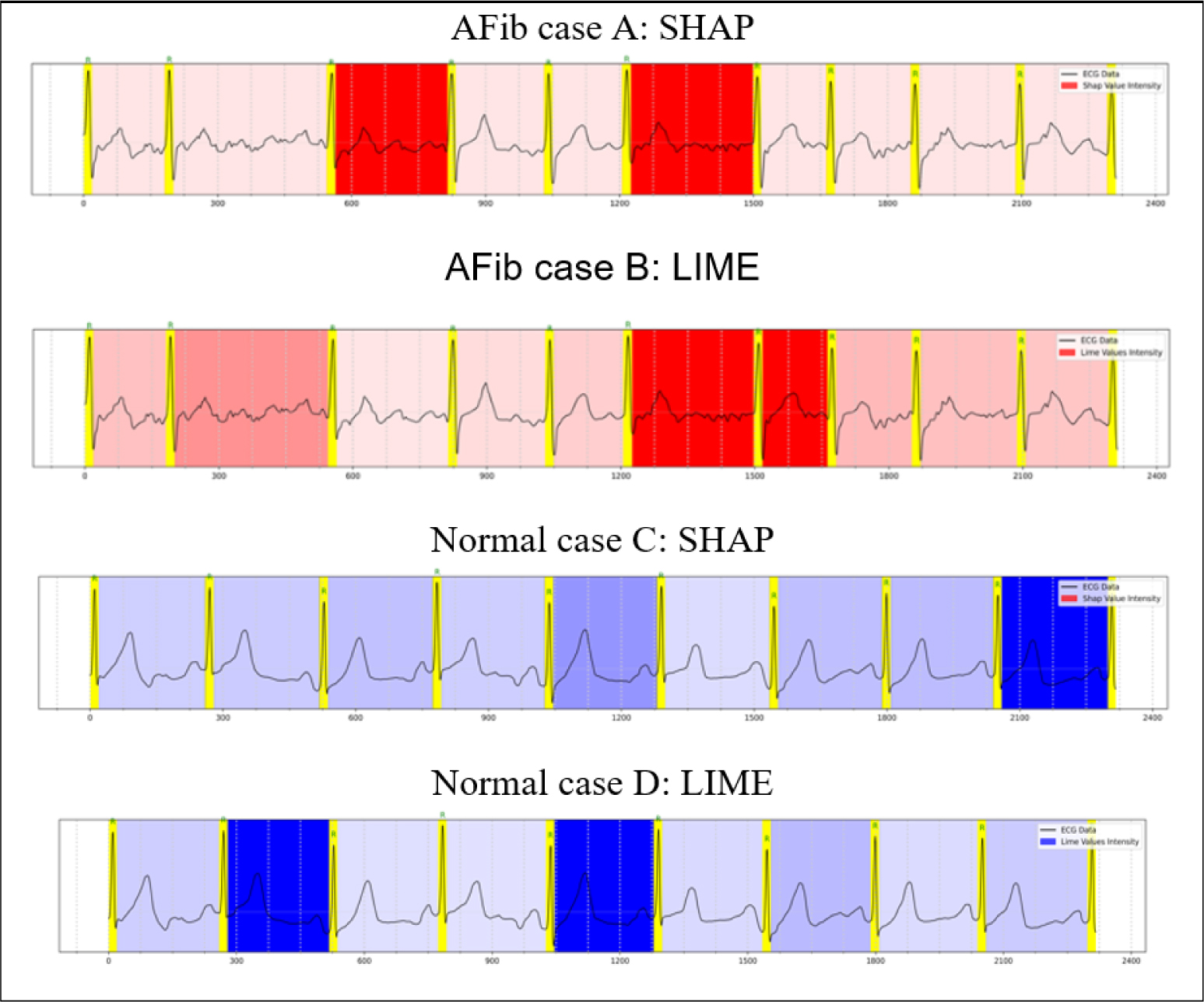
Aggregated RR-interval importance generated by SHAP and LIME.

This limitation of directly applying LIME and SHAP values on ECGs suggests the need for a more clinically-aligned interpretation that can analyze ECG signals at a more appropriate temporal scale and incorporate domain-specific knowledge about cardiac rhythm patterns. To address this challenge, we introduced a new method for enhancing the interpretability of SHAP and LIME values by aggregating the values with critical clinical markers, the RR interval (See Figure 2). By accumulating LIME or SHAP values across these intervals, we created a more intuitive visualization that mirrors the clinical approach to ECG analysis. (See Figure 6)

This aggregation transforms the point-wise importance values into meaningful beat-level insights, where each RR interval receives a composite score based on its contained LIME or SHAP values. The resulting visualization provides an intuitive representation of the model’s decision-making process: intervals shown in deeper red indicate stronger evidence for AFib, while blue regions suggest normal rhythm characteristics. Yellow markers highlight the R peaks, serving as reference points for beat delineation. This enhanced visualization approach not only preserves the quantitative rigor of LIME and SHAP analyses but also presents the information in a format that resonates with clinicians’ diagnostic workflows, effectively bridging the gap between AI interpretability and clinical practice.

### 3.3 Beat-level localized XAI Analysis for AF Prediction

To gain deeper insight into the beat-level characteristics, we conducted a detailed analysis of the top beats identified by our LIME/SHAP RR-interval aggregation method. This focused examination revealed two clinically significant patterns that distinguish AFib from normal rhythms.

**Figure 7:**
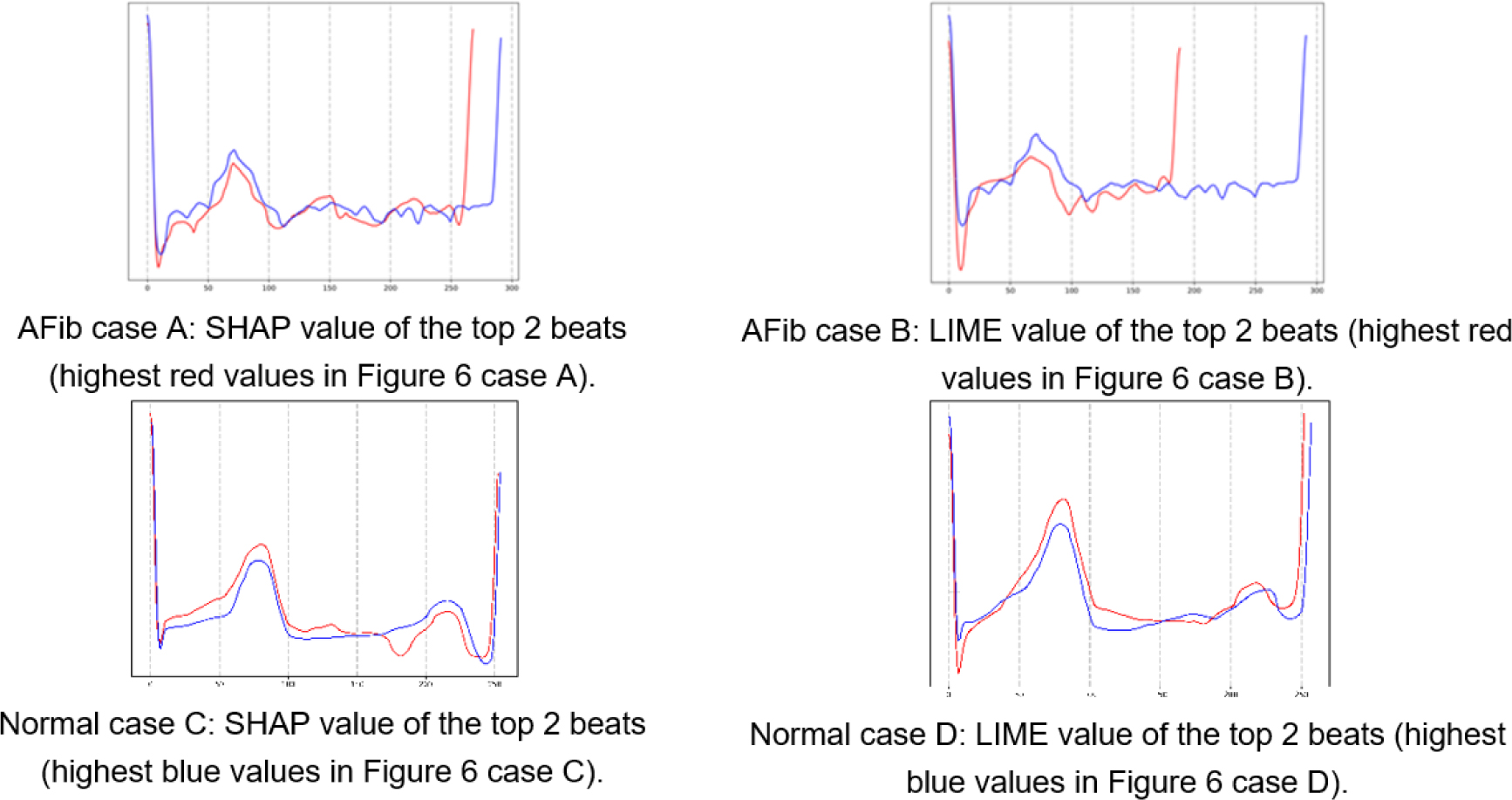
Examination of beat-level patterns after LIME and SHAP annotation.

First, in AFib cases (top row of Figure 7), there is a notable absence of the P-wave preceding the R peak, which is consistent with the pathophysiology of atrial fibrillation where coordinated atrial depolarization is disrupted. In contrast, the normal cases (bottom row) display clear, well-defined P-waves before each QRS complex, indicating normal atrial electrical activity. Second, our analysis confirmed irregular RR intervals in AFib cases, manifesting as inconsistent spacing between consecutive R peaks, whereas normal cases demonstrated more uniform RR intervals. These observations align precisely with established clinical criteria for AFib diagnosis and validate that our XAI approach successfully captures the most clinically relevant features for rhythm classification. The ability of our method to highlight these specific morphological and temporal characteristics demonstrates its potential as a valuable tool for supporting clinical interpretation of AI-based ECG analysis.

**Figure 8:**
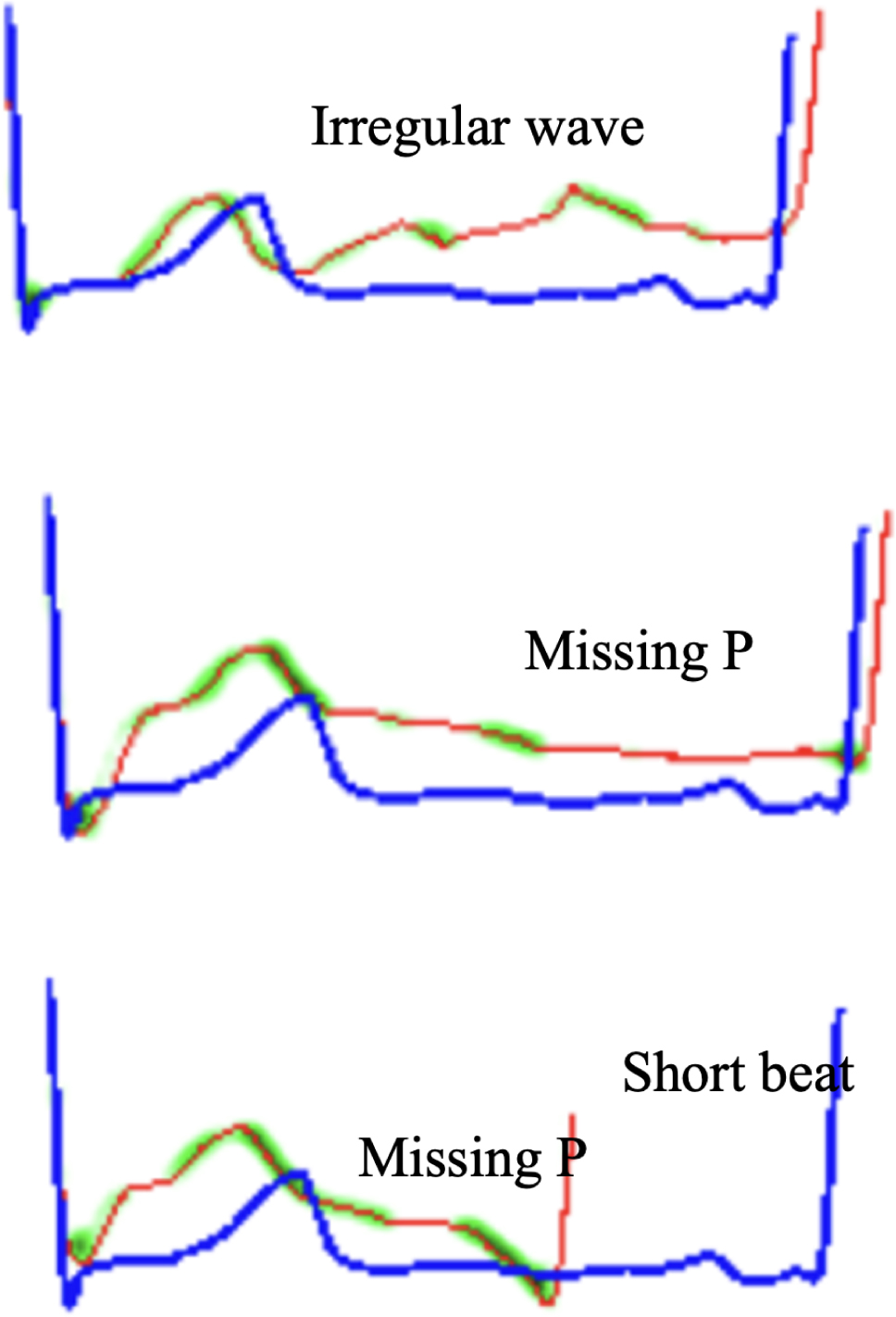
Grad-CAM predicted localized regions for AFib ECGS.

The beat-level patterns motivated us to explore more sophisticated methods for localized XAI of AFib predictions. We developed a Grad-CAM-based method, which provides visual explanations through gradient-based localization of deep network features. As shown in Figure 8, The Grad-CAM method can highlight specific regions within the ECG beats that were most influential in AFib classification. In these visualizations (Figure 8), blue waveforms represent normal ECG morphology within RR-intervals, while red waveforms indicate AFib patterns. The green-highlighted regions identify areas that Grad-CAM determined to be most significant for AFib detection. The results revealed key morphological features, for example 1) the absence of prominent P-waves in the pre-QRS complex region, 2) inconsistent short or long beats, and 3) irregular waveform patterns between RR-intervals. These Grad-CAM visualizations provide clinically meaningful localization of diagnostic features, which complement LIME and SHAP methods. Togher, these methods offer comprehensive multi-level clinically interpretable explanations that align with established criteria for AFib diagnosis.

## 4 Conclusion

This study makes significant contributions to the field of AI-assisted cardiac diagnosis by introducing a comprehensive multi-level XAI framework for AFib detection. Our innovative RR-interval aggregation method successfully bridges the gap between granular AI interpretations and clinical diagnostic patterns, transforming traditional LIME and SHAP analyses into clinically meaningful insights. The integration of Grad-CAM further enhances this framework by providing precise localization of diagnostic features, successfully identifying key AFib markers such as P-wave absence, T-wave abnormalities, and irregular RR intervals. The high performance of our model (91.3% precision for AFib detection and 98.4% for normal rhythm) demonstrates that interpretability can be achieved without sacrificing accuracy. Importantly, our approach aligns AI explanations with established clinical diagnostic criteria, potentially accelerating the adoption of AI tools in cardiac care. While our results are promising, future work should focus on validating these methods across diverse patient populations and clinical settings. The success of this multi-level XAI framework suggests a promising direction for developing interpretable AI systems in healthcare, where transparency and clinical relevance are paramount for practical implementation.

## Figures and Tables

**Table 1:** Evaluation results of the developed Deep Learning model for classifying ECGs for Afib

	Precision	Recall	F1-Score

AFib	0.913	0.887	0.900
Normal	0.984	0.988	0.986
